# Correction: Split-anion solvent extraction of light rare earths from concentrated chloride aqueous solutions to nitrate organic ionic liquids

**DOI:** 10.1039/d0ra90130j

**Published:** 2020-12-15

**Authors:** Mercedes Regadío, Tom Vander Hoogerstraete, Dipanjan Banerjee, Koen Binnemans

**Affiliations:** KU Leuven – University of Leuven, Department of Chemistry Celestijnenlaan 200F, P. O. Box 2404 3001 Heverlee Belgium koen.binnemans@kuleuven.be; Dutch-Belgian Beamline (DUBBLE), ESRF – the European Synchrotron CS 40220 F-38043 Grenoble Cedex 9 France

## Abstract

Correction for ‘Split-anion solvent extraction of light rare earths from concentrated chloride aqueous solutions to nitrate organic ionic liquids’ by Mercedes Regadío *et al.*, *RSC Adv.*, 2018, **8**, 34754–34763, DOI: 10.1039/c8ra06055j.

The authors regret that an incorrect figure caption was given for Fig. 5. The correct version is presented below.

**Fig. 5:**
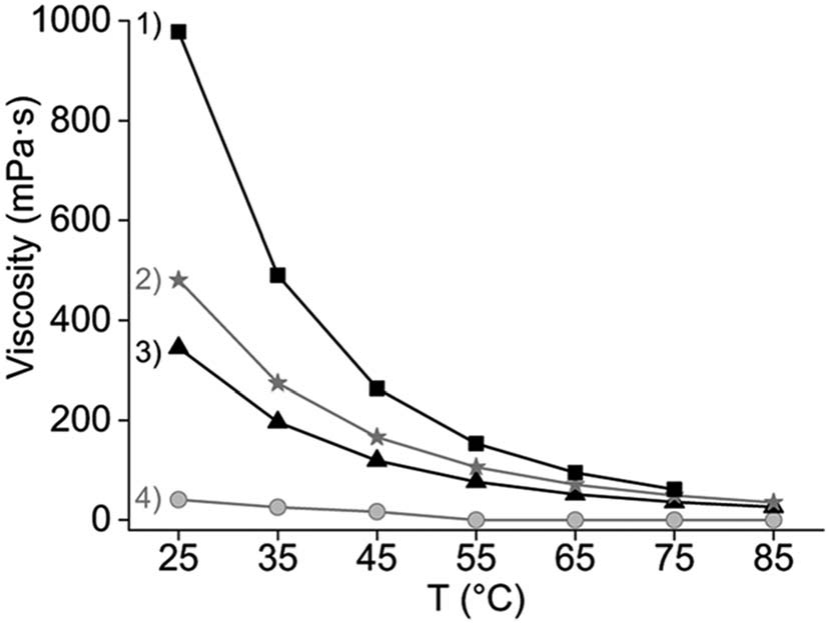
Viscosity as a function of the temperature and the organic phase composition: (1) after loading 39 g L^−1^ of REE in 20 v% Cy923 in [C101][NO_3_], (2) pure [C101][NO_3_], (3) 20 v% Cy923 in [C101][NO_3_] and (4) pure Cy923.

The Royal Society of Chemistry apologises for these errors and any consequent inconvenience to authors and readers.

## Supplementary Material

